# Influence Pattern and Mechanism of Increased Nitrogen Deposition and AM Fungi on Soil Microbial Community in Desert Ecosystems

**DOI:** 10.3390/microorganisms13122660

**Published:** 2025-11-22

**Authors:** Hui Wang, Wan Duan, Qianqian Dong, Zhanquan Ji, Wenli Cao, Fangwei Zhang, Wenshuo Li, Yangyang Jia

**Affiliations:** 1 College of Ecology and Environment, Xinjiang University, Urumqi 830046, China; 2 Key Laboratory of Oasis Ecology, Xinjiang University, Urumqi 830046, China

**Keywords:** nitrogen deposition, Arbuscular mycorrhizal fungi, soil microbial community, phospholipid fatty acid, Gurbantunggut Desert

## Abstract

With continuous increases in nitrogen (N) deposition in the future, its impacts on terrestrial ecosystems are attracting growing concern. Arbuscular mycorrhiza (AM) fungi play a crucial role in shaping both soil microbial and plant communities. AM fungi play a crucial role in shaping the soil microbial and plant communities, yet their patterns of influence under increased N deposition scenarios remain unclear, particularly in desert ecosystems. Therefore, we conducted a field experiment simulating increased N deposition and AM fungal suppression to assess the effects of increased N deposition and AM fungi on soil microbial communities, employing phospholipid fatty acid (PLFA) biomarker technology in the Gurbantunggut Desert of Xinjiang. We found that increased N deposition promoted soil microbial biomass, including AM fungi, fungi, Actinomycetes (Act), Gram-positive bacteria (G^+^), Gram-negative bacteria (G^−^), and Dark Septate Endophyte (DSE). AM fungal suppression significantly increased the content of soil Act and G^+^. There were clearly and significantly interactive effects of increased N deposition and AM fungi on soil microbial contents. Both increased N deposition and AM fungi caused significant changes in soil microbial community structure. Random forest analysis revealed that soil nitrate N (NO_3_^−^-N), Soil Organic Carbon (SOC), and pH were main factors influencing soil microorganisms; soil AM fungi, G^+^, and Act significantly affected plant Shannon diversity; soil G^−^, Act, and fungi posed significant effects on plant community biomass. Finally, the structure equation model results indicated that soil fungi, especially AM fungi, were the main soil microorganisms altering the plant community diversity and biomass under increased N deposition. This study reveals the crucial role of AM fungi in regulating soil microbial responses to increased N deposition, providing experimental evidence for understanding how N deposition affects plant communities through soil microorganisms.

## 1. Introduction

Human activities are accelerating global climate change with regional climate shifts, extreme precipitation events, and increased nitrogen (N) deposition [1]. The sharp increase in atmospheric N deposition caused by anthropogenic factors is one of the critical factors affecting terrestrial ecosystems, particularly in Asia [2]. Increased N deposition increases soil N content and is a major determinant of soil microbial community structure and functions [3,4,5]. Soil microorganisms, as a crucial component of the belowground ecosystem, play vital roles in maintaining diverse ecosystem functions [6]. Previous studies have indicated that increased N deposition generally increased soil microbial biomass, which may be attributed to the limited nitrogen nutrient for soil microbial growth [7,8]. Furthermore, above and below-ground components form a unified organic entity, where changes in soil microbial community structure and diversity can influence above-ground plant communities [9]. There is mounting evidence that increasing N deposition represents a significant indicator of global change and highly affects soil and plant community structure by altering the dominance of certain species [10,11]. However, compared to other ecosystems, increased N deposition could cause more considerable changes in the soil microbial community of desert ecosystems. This is because N is the critical limiting factor for both plant growth and ecosystem functions in these regions, warranting an urgent in-depth investigation.

As a global ecological driver, increased N deposition poses significant impacts on soil microbial communities, while the impacts differ across different ecosystems. Prior studies have indicated that increased N deposition has a significant impact on the soil bacterial community structure, but only a minimal effect on the soil fungal community structure in shrubland ecosystems [12,13]. However, previous research has demonstrated that increased N deposition exerts a limited impact on the structure of the soil microbial community in grassland ecosystems [14,15], but highly altered the soil fungal community in one forest ecosystem [16]. Previous studies on the effects of increased N deposition on soil microbial communities have primarily focused on its impact on soil bacterial or fungal communities. However, this approach might have overlooked other soil microorganisms, such as Actinomycetes (Act), Gram-positive bacteria (G^+^) and Gram-negative bacteria (G^−^), resulting in biases regarding the responses of the soil microbial community to increased N deposition. Our study found that increased N deposition promoted the growth of soil Act, G^−^ and fungi. Furthermore, increased N deposition affects the soil microbial community by enhancing nutrient availability and leading to soil acidification [17]. It is widely accepted that increased N deposition reduces the soil microbial diversity through the reduction in soil pH induced by chronic excess N deposition [18]. Meanwhile, a recent study revealed that the effect of increased N deposition on the soil bacterial and fungal communities was primarily mediated by the rise in soil nitrogen content [19]. The typical characteristics of desert ecosystems are the lack of soil nutrients and excessively high soil pH. [20]. The response of the soil microbial community to increased N deposition may contrast with that observed in other ecosystems. Firstly, desert ecosystems have low soil N availability, meaning that the soil microbial communities are more restricted by N [8]. Thus, increased N deposition could have strong effects on the soil microbial community with those soil microorganisms that are fast-growing [21]. Secondly, compared to other ecosystems, soil microorganisms in desert ecosystems are likely already adapted to the barren environment. Consequently, these microbial communities may possess a high tolerance to changes in soil nutrients and pH [22]. Although previous studies have reported varying responses of the soil microbial communities to increased N deposition across different ecosystems, the underlying mechanisms in desert ecosystems remain unclear.

Arbuscular mycorrhiza (AM) fungi show tight and complex synergies with other soil microorganisms and constitute a critical component of soil microbial communities [23]. Previous study indicated that the presence of AM fungi increased the relative abundance of soil fungi and G^−^, thereby altering soil microbial community structure [24]. Inoculation with AM fungi can enhance species richness of both soil bacteria and fungi [25]. Research further indicated that AM fungi can increase total soil microbial biomass by 6.3%, bacterial biomass by 20.4%, and fungal biomass by 45.0% [26]. These results indicate the positive effects of AM fungi on other soil microorganisms, while there are also studies which revealed that AM fungi could compete with other fungal types for resources, thereby inhibiting their growth and reproduction [27,28]. Furthermore, AM fungi directly interconnect plant roots with the soil microbial communities via their hyphal networks, forming a plant-AM fungi-bacteria continuum [29]. The effects of AM fungi on the soil microbial community are highly affected by plants. Under increased N deposition, improved soil nutrient availability decreases plant dependence on AM fungi. This reduction leads to a decreased biomass and diversity of the AM fungal community, subsequently altering the soil microbial community through cascade effects [30]. Increased N deposition affects plant communities by altering the soil microbial communities, while AM fungi regulate both the plant and soil microbial communities [31]. However, how increased N deposition and AM fungi interact to affect plant communities through altering the soil microbial community remains unclear, particularly in desert ecosystems. Therefore, it is very important to explore the combined effects of increased N deposition and AM fungi on the soil microbial community.

Global desert ecosystems are approximately 36 million km^2^, accounting for 24% of the Earth’s land area [32]. In China, desert ecosystems cover more than one fourth of the country’s land area [33]. Notably, the desert area in Xinjiang with the largest desert and arid land accounts for 41% of China’s desert area [34]. Temperate desert ecosystems with barren environments, characterized by high temperature, low precipitation, nutrient-poor soils, and sparse plant coverage, are the most fragile terrestrial ecosystems [20]. They are particularly sensitive to altered precipitation and increased N deposition. The Gurbantunggut Desert in Xinjiang, with its unique geographical location and climatic conditions, serves as a representative area for studying the impacts of climate change on desert ecosystems. Recent observations revealed that the climate in the Gurbantunggut desert presents a “warm and wet” trend, and the atmospheric N deposition has continuously increased in the past two decades [35,36]. Therefore, we conducted an in situ experiment under the context of increased precipitation. By simulating enhanced N deposition and inhibiting AM fungal activity, this study reveals how the soil microbial communities respond to their interactions and the underlying mechanisms involved. Our main objectives were as follows: (1) To explore the interactive effects of increased N deposition and AM fungi on soil microbial community; (2) to uncover the influence pattern of increased N deposition and AM fungi on the plant community through soil microorganisms.

## 2. Materials and Methods

### 2.1. Study Site

This study was conducted at the southern edge of the Gurbantunggut Desert. As the second largest desert in China, it is located in the center of the Junggar Basin in northern Xinjiang (44° N–47° N, 85° E–90° E), covering a total area of approximately 48,800 square kilometers. The study area has a typical temperate continental arid climate, with an average annual temperature of 6–10 °C and an average annual precipitation of 215.6 mm, but the annual evaporation exceeds 2000 mm [37]. The soils are classified as gray desert soil based on the Chinese soil classification system, with aeolian sand dominating the composition from the surface down to a depth of 100 cm. The soil has poor nutrient content (such as phosphorus and nitrogen), with relatively high bulk density (1.58 ± 0.1 g/cm^3^) and strongly alkaline reaction (pH 9.55 ± 0.14) [38]. The snow cover reaches a thickness of 20–30 cm in winter, and spring snowmelt significantly increases soil moisture [39]. In early spring, ephemeral plants can use the favorable soil water, finish their life cycle within 60 to 70 days, and dominate as the predominant species with coverage reaching over 60% in early spring [40,41].

### 2.2. Experimental Design

The experiment was initiated in 2014. Prior to the experiment, we selected a suitable study area in the Gurbantunggut Desert and erected a perimeter fence around it to prevent trampling by surrounding animals. The experiment used a two-factor (i.e., increased N deposition and AM fungi) randomized complete block design with four treatments. Based on projected precipitation changes in the study area, precipitation is expected to fluctuate with an overall increase of 40 mm over the next 50–100 years [42]. Therefore, to simulate future precipitation changes, a uniform 40 mm water addition was implemented across all experimental treatments in this study. The control treatment was only supplemented with a 40 mm increase in precipitation (W); benomyl was applied with water to reduce the AM fungal activity (BW); additional N was applied with water to simulate increased N deposition (NW); finally, benomyl application plus N addition with water was applied in the last treatment (BNW). Benomyl was selected to suppress the activity of AM fungi. Substantial evidence has demonstrated that under natural conditions, this agent effectively reduces AM fungal colonization without altering other soil microbial communities and has negligible effects on non-target fungi within the same area. [43,44,45]. Over the past three decades, increased N deposition in this study area has reached 1.58 g/m^2^/year due to human activities and industrial development [42]. As predicted by Galloway et al., the global increased N deposition is projected to double by 2050 [46]. Therefore, we established the N addition treatment with 3 g/m^2^/yr to simulate the future N deposition rate at the experimental site.

Before the germination of desert ephemeral plants, we established 24 plots measuring 2.5 × 2.5 m, with a 2 m buffer zone between any two adjacent plots. Each large plot was subdivided into four 1 × 1 m^2^ subplots, with each plot corresponding to one treatment and each treatment having six replicates. One subplot was randomly selected annually for observation and sampling. In the N addition treatment, to simulate N deposition, ammonium nitrate (NH_4_NO_3_) was dissolved in 10 L of water and uniformly sprayed onto the plots every two weeks during the growing season using a portable sprayer. The total annual N input was administered in four equal applications, i.e., 0.75 g/m^2^ each time. For benomyl application, benomyl was applied in aqueous solution since water is required as the carrier medium for soil infiltration to suppress AM fungal activity. Specifically, we dissolved benomyl in the same water volume (10 L of water, 0.6 g active ingredient per liter of water). The control treatment (W) received 10 L of water without N and benomyl, and the treatments were applied four times annually from 10 March to 10 May.

### 2.3. Sampling Protocol

Plant samples were collected at the peak plant community biomass on 25 May 2016 and 2017, respectively. The above-ground net primary productivity (ANPP), plant coverage and density were measured for each treatment. Each plant was taxonomically identified and oven-dried at 65 °C for 72 h, weighed, and the dry weight of each species was recorded to calculate the total ANPP.

After plant sampling and removing dead leaves from soil surface, soil samples collected at three random sampling points were selected within the plot area of each treatment. We collected topsoil samples using a 5 cm diameter soil auger at a depth of 20 cm, sieved the samples from each treatment through a 2 mm mesh, and then transported them to the laboratory. In total, 24 composite samples per year (4 treatments × 6 replicates), for a total of 48 samples in two years. Each soil sample was then divided into four subparts: one part was air-dried at room temperature for soil physicochemical properties analysis, including pH, Soil Organic Carbon (SOC), and Available Phosphorus (AP); the second part was stored at 4 °C for the determination of microbial biomass carbon (MBC) and microbial biomass nitrogen (MBN) within one month; the third part was stored at −20 °C for the measurement of nitrate N (NO_3_^−^-N) and ammonium N (NH_4_^+^-N); and the remaining portion was preserved at −80 °C for the soil microbial community analysis using phospholipid fatty acid (PLFA) profiling. Utilizing the correspondence between different PLFA markers and specific microbial groups is more conducive to explaining changes in the community structure [47].

### 2.4. Measurement and Calculation of Soil Microbial Community and Soil Parameters

The determination of soil microbial PLFA includes bacteria, fungi, Act, G^+^, G^−^, Dark Septate Endophytes (DSE), and AM fungi. The extraction method of PLFA in the soil is based on the research of Bossio and Scow [48]. For the determination of PLFA content, eight grams of freeze-dried soil samples were accurately weighed and subjected to two rounds of oscillatory extraction using a mixture of citrate buffer (0.15 M, pH 4.0, Sigma-Aldrich B9434; Merck KGaA, Darmstadt, Germany), chloroform, and methanol in a ratio of 0.8:1:2. The fatty acid fractions were then separated via a silica solid-phase extraction column, and alkaline methanolysis of the phospholipid fatty acids was carried out with methyl nonadecanoate (19:0, Sigma CRM47885; Merck KGaA, Darmstadt, Germany, final concentration 10 μM) as the internal standard. The fatty acid methyl esters obtained were separated, quantified, and identified using gas chromatography (GC). The qualitative and quantitative analysis of fatty acids was conducted using a gas chromatograph (GC-FID, Agilent 7890B, Santa Clara, CA, USA) from Agilent Technologies (Palo Alto, CA, USA) and the MIDI Sherlock Microbial Identification System from MIDI, Inc. (Newark, DE, USA) [49]. The PLFA analysis was conducted according to the method of Bossio and Scow, and the fatty acids were classified into six categories: G^+^ (i12:0, i13:0, i14:0, a15:0, i15:0, i16:0, a16:0, a17:0, i17:0, i18:0), G^−^ (i15:0 3OH, 16:1ω7c, 16:1ω9c, 17:1ω7c, 17:1ω8c, i17:0 3OH, 18:1ω7c, cy17:0), Act (10Me16:0, 10Me17:0, 10Me18:0), fungi (18:1ω9c, 18:2ω6,9, 18:3ω6c(6,9,12)), AM fungi (16:1ω5c), DSE (16:00:00, 18:00:00). The mass of the specific fatty acid FA16:1ω5c of AM fungi is considered to be the biomass of AM fungi [49].

By calculating the Shannon diversity index (H), Simpson’s dominance index (D), and Pielou’s evenness index (J), the soil microbial diversity is characterized. The Shannon diversity index (H) was calculated as $H=-\sum_{i=1}^{S}{(P}_{i}lnP_{i})$; Simpson’s dominance index (D) was calculated as $D=1-\sum_{i=1}^{S}P_{i}^{2}$; and Pielou’s evenness index (J) was calculated as $J=\frac{H}{lnS}$. In the formulas, $P_{i}$ represents the proportion of individuals of the i-th species to the total number of individuals, calculated as $P_{i}$ = ni/N, where ni is the number of individuals of the i-th species, and N is the total number of individuals of all species; S is the total number of species in the community.

Mix the soil with deionized water at a 1:5 soil-to-water ratio, filter the supernatant, and measure the soil pH using a pH meter (Seven Easy, Mettler Toledo, Greifensee, Switzerland). Soil organic carbon was measured by the oxidation method with a potassium dichromate-sulfuric acid solution. Soil NO_3_^−^-N and NH_4_^+^-N were determined using a continuous flow analyzer (AA3, SEAL Analytical, Southampton, UK; formerly Bran + Luebbe Corp). Soil microbial biomass carbon and nitrogen were extracted by chloroform fumigation followed by K_2_SO_4_ leaching. Mycelial density was determined using the membrane filtration method, where the intersection points between hyphae and grid lines were counted by the grid-line intersect method, followed by calculation of mycelial density using a specific formula [50]. Spore density was assessed through wet sieving and decanting combined with sucrose density gradient centrifugation, with subsequent microscopic counting of spores in the collected suspension [51].

### 2.5. Data Statistics and Analysis

We performed analysis of variance (ANOVA) using SPSS 19.0 (SPSS Inc., Chicago, IL, USA) with all data tested for normality and homogeneity of variance priorly. Firstly, a two-way ANOVA was conducted to evaluate the main effects and interaction between increased N deposition and AM fungi on the following variables: changes in soil physicochemical properties, PLFA content of various soil microorganisms, and the diversity indices of soil microbial and plant communities. Secondly, non-metric multidimensional scaling (NMDS) based on Euclidean distance was employed to compare the compositional and structural differences in the soil microbial communities under increased N deposition and AM fungi treatments. Correlation heatmaps were applied to illustrate the complex relationships among soil physicochemical properties, PLFA content of each soil microorganism, and diversity index of the soil microbial and plant communities. Redundancy Analysis (RDA) was utilized to demonstrate the fitted values of multiple multivariate linear regressions between response and explanatory variables involving soil physicochemical properties, PLFA content of each soil microorganisms, and plant community. Random forest models were further established to separately analyze the effects of soil physicochemical properties on PLFA content of each soil microorganism, as well as the influence of PLFA content of each soil microorganism on the plant community diversity and biomass, identifying key influence factors on soil microorganisms and the plant community. Finally, to estimate the potential pathways through which increased N deposition and AM fungi affect the plant community via soil microorganisms, structural equation modeling (SEM) was conducted. SEM, RDA and correlation model construction were all implemented using the R version 4.3.3. This study employed multiple indices to evaluate the goodness-of-fit of the structural equation model. The results demonstrated a good model-data fit: PVALUE > 0.05, RMSEA = 0.06 (90% CI: 0.030–0.065).

## 3. Results

### 3.1. Increased N Deposition and AM Fungi Significantly Altered Soil Physiochemical Properties and Plant Community Composition

Our study found that increased N deposition posed significant effects on soil physicochemical properties (Table 1 and Table S1). Increased N deposition led to a rise in soil NO_3_^−^-N and NH_4_^+^-N concentrations, while significantly reducing soil pH and available phosphorus content (Table 1 and Table S1). Meanwhile, AM fungal suppression had limited effects on soil physicochemical properties. Increased N deposition and AM fungi had a significant interactive effect on soil physicochemical properties. (Table 1 and Table S1). When AM fungi were present, increased N deposition significantly reduced soil pH and MBN, and increased SOC, while, when AM fungi were suppressed, no significant changes were detected in soil pH, MBN, or SOC content (Table 1 and Table S1).

**Figure 1:**
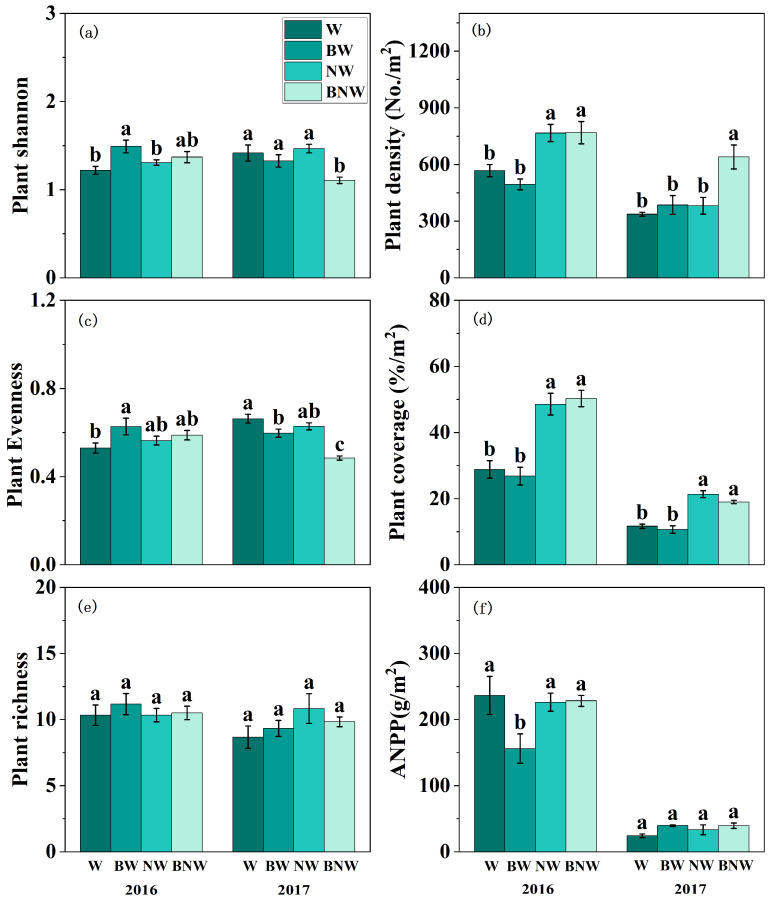
The effects of increased nitrogen deposition and AM fungi on plant community PLFA content in 2016 and 2017. All data are presented as “mean ± standard error”. Note: Different lowercase letters (a, b, c) indicate significant differences among treatments. (*p* < 0.05). W, water treatment; BW, benomyl + water treatment; NW, N + water treatment; BNW, benomyl + N + water treatment. (**a**) Shannon diversity under different treatments; (**b**) Plant density under different treatments; (**c**) Plant Evenness under different treatments; (**d**) Plant coverage under different treatments; (**e**) Plant richness under different treatments; (**f**) ANPP under different treatments. Results of the ANOVA are detailed in Supplementary Table S2.

For the plant community, our results revealed that increased N deposition significantly enhanced plant community density and plant coverage, but decreased plant community evenness (Figure 1b–d). AM fungal suppression reduced ANPP in 2016, and decreased plant community evenness in 2017 (Figure 1; Table S2). Significant interactive effects of increased N deposition and AM fungi on plant community were also observed (Figure 1; Table S2). When AM fungi were present, increased N deposition showed no significant effects on plant density or ANPP; while, when AM fungi was suppressed, increased N deposition markedly increased both plant density and ANPP, particularly in 2017 (Figure 1). As for Shannon diversity and community evenness, increased N deposition showed no significant impacts when AM fungi were present but significantly reduced both Shannon diversity and evenness when AM fungi were suppressed (Figure 1a,c).

### 3.2. Increased N Deposition and AM Fungi Influenced Soil Microbial Communities

**Figure 2:**
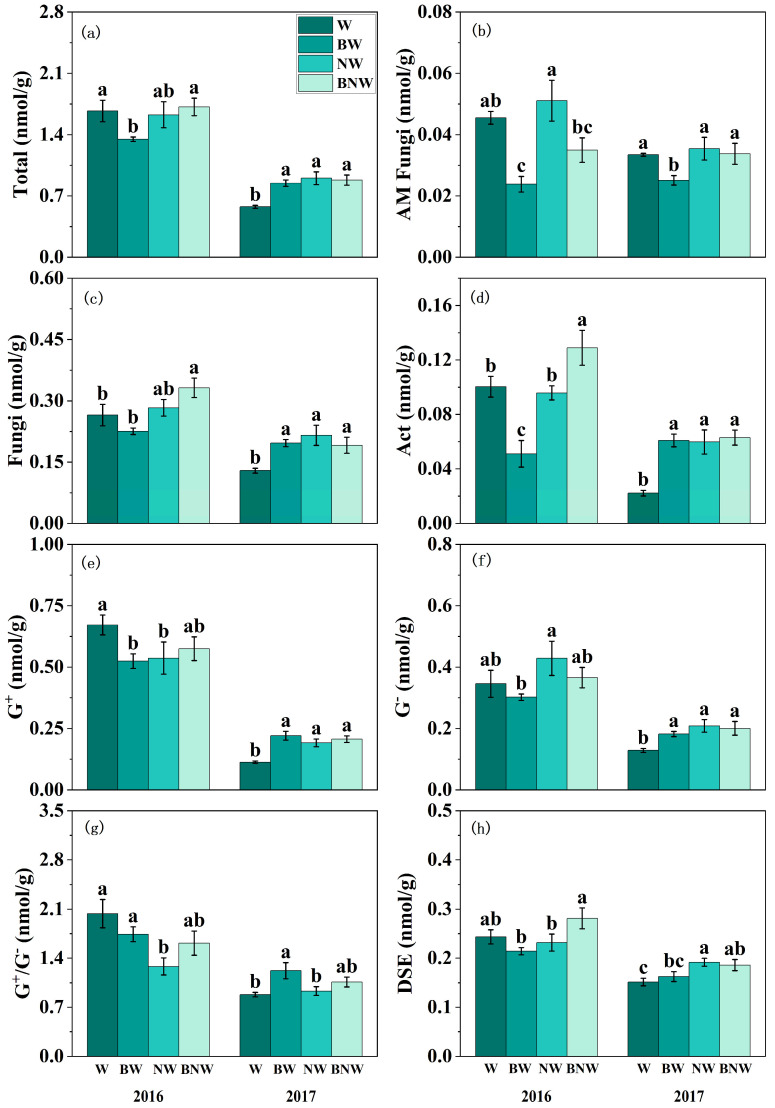
The effects of increased nitrogen deposition and AM fungi on microbial community PLFA content in 2016 and 2017. All data are presented as “mean ± standard error”. Note: Different lowercase letters (a, b, c) indicate significant differences among treatments. (*p* < 0.05). Total: Total soil microbial biomass; AM Fungi: Arbuscular mycorrhizal fungi; Act: Actinomycetes; G^+^: Gram-positive bacteria; G^−^: Gram-negative bacteria; G^+^/G^−^: Ratio of Gram-positive to Gram-negative bacteria; DSE: Dark septate endophytes. (**a**) Total soil microbial biomass assessed by PLFA under different treatments; (**b**) AMF biomass assessed by PLFA under different treatments; (**c**) Fungi biomass assessed by PLFA under different treatments; (**d**) Act biomass assessed by PLFA under different treatments; (**e**) G^+^ biomass assessed by PLFA under different treatments; (**f**) G^−^ biomass assessed by PLFA under different treatments; (**g**) G^+^/G^−^ biomass assessed by PLFA under different treatments; (**h**) DSE biomass assessed by PLFA under different treatments. Results of the ANOVA are detailed in Supplementary Table S3.

The study found that increased N deposition exerted a significant influence on the soil microbial community, with the most pronounced effects observed in 2017 (Figure 2; Table S3). Increased N deposition clearly promoted soil AM fungi, fungi, Act, G^+^, G^−^, DSE, and total soil microbial biomass, which were more pronounced in 2017 (Figure 2; Table S3). AM fungal suppression significantly increased the content of soil Act and G^+^ (Figure 2d,e; Table S3). Moreover, there were clearly and significantly interactive effects of increased N deposition and AM fungi on soil microbial contents (Table S3). For example, when AM fungi were suppressed, increased N deposition significantly increased the PLFA content of soil fungi and Act; while posed limited effects when AM fungi were present, especially in 2016 (Figure 2c,d). For G^+^ and G^−^, increased N deposition significantly increased their PLFA content when AM fungi were present, while no significant effect was observed when AM fungi were suppressed (Figure 2e,f). This pattern was particularly notable in 2017 (Table S3). Notably, corresponding variations were observed in spore density and hyphal density, as well as in the soil AM fungal PLFA content under increased N deposition and AM fungal treatments (Figure S1 and Figure 2b).

**Figure 3:**
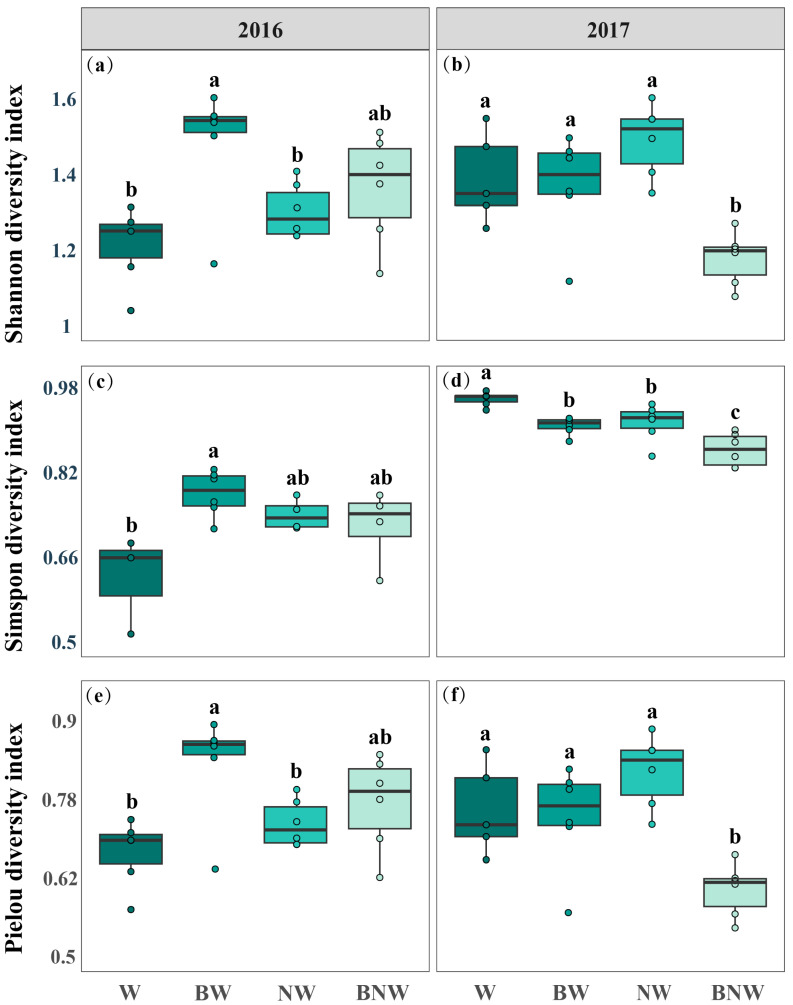
Effects of increased nitrogen deposition and AM fungi on soil microbial community diversity in 2016 (**a**,**c**,**e**) and 2017 (**b**,**d**,**f**). All data are presented as mean ± standard error. Note: Different lowercase letters (a, b, c) indicate significant differences among treatments. (*p* < 0.05). ANOVA results are shown in Table S4.

Increased N deposition and AM fungi both had an impact on the soil microbial community diversity over the two-year period (Figure 3). The results of ANOVA for microbial community diversity are presented in the Supplementary Materials (Table S4). AM fungal suppression increased Shannon diversity, Simpson diversity, and Pielou’s evenness of the soil microbial community, but increased N deposition posed limited effects in 2016 (Figure 3a,c,e). In contrast, increased N deposition decreased Shannon diversity, Simpson diversity, and Pielou’s evenness of the soil microbial community, but AM fungal suppression posed limited effects in 2017 (Figure 3b,d,f). There were significant interactive effects of increased N deposition and AM fungal suppression on the soil microbial community (Figure 3). Furthermore, our results found that increased N deposition suppressed the content of AM fungi and G^−^ bacteria, while AM fungal suppression had no effects on G^−^ (Figure 2). Without increased N deposition treatment, AM fungal suppression significantly increased the content of G^+^ bacteria, but no significant effect was observed under increased N deposition. An NMDS analysis revealed that increased N deposition and AM fungal suppression strongly affected soil microbial community structure (Figure S3). These results indicated that increased N deposition and AM fungal suppression interactively affected the soil microbial community by jointly altering the PLFA content of soil microorganisms.

### 3.3. Soil Physicochemical Properties, Soil Microbial and Plant Community Properties Complexly Interacted

**Figure 4:**
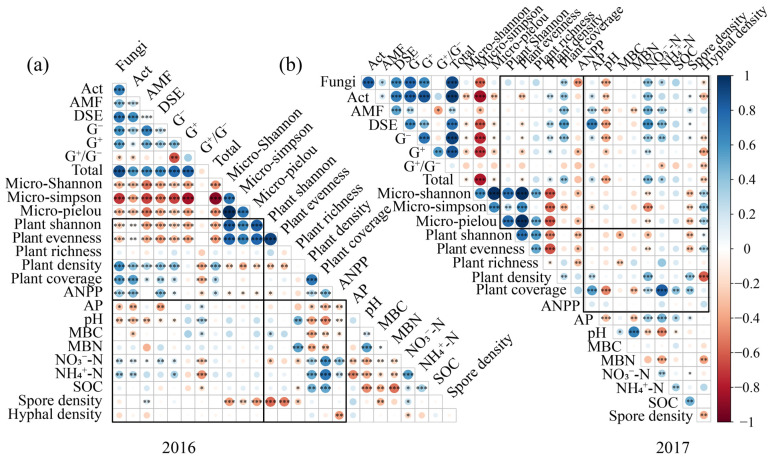
Correlation analysis of soil microbial PLFA content, plant PLFA content, and soil physicochemical factors in 2016 (**a**) and 2017 (**b**). Note: AP, Available phosphorus; SOC, Soil organic carbon; NO_3_^−^-N, Nitrate nitrogen; NH_4_^+^-N, Ammonium nitrogen; MBC, Microbial biomass carbon; MBN, Microbial biomass nitrogen. Statistical significance was defined as follows: * *p* < 0.05 was considered significant, ** *p* < 0.01 was considered highly significant, and *** *p* < 0.001 was considered extremely significant.

The correlation heatmap analysis revealed that there were tight and complex relationships among the soil physicochemical properties, soil microbial communities, and plant communities (Figure 4). For soil physicochemical properties, soil pH, NO_3_^−^-N and NH_4_^+^-N showed close relationships with soil microbial community and plant community (Figure 4). Soil pH showed significantly negative relationships with soil fungi, Act, and AM fungi, while soil NO_3_^−^-N and NH_4_^+^-N were significantly and positively correlated with soil fungi, Act, and G^+^ (Figure 4). Soil NO_3_^−^-N and NH_4_^+^-N exhibited significantly positive correlations with plant density and coverage, while soil pH and MBC showed significantly negative correlations with plant density and coverage (Figure 4). Furthermore, soil microorganisms showed close relationships with the plant community, particularly in 2016 (Figure 4). Soil fungi and Act showed significant and positive relationships with plant density, coverage, and ANPP, while soil Act and DSE showed significant and negative relationships with plant Shannon diversity and community evenness, which were more pronounced in 2016. The RDA results further confirmed that soil NO_3_^−^-N, NH_4_^+^-N, and AP showed significant effects on soil microorganisms (Figure S4a,b). Soil pH, NO_3_^−^-N, NH_4_^+^-N, and SOC exhibited significant influences on plant community (Figure S4c,d). And, soil fungi and AM fungi were the top two factors influencing plant community (Figure S4e,f).

### 3.4. Underlying Influence Mechanisms of Increased N Deposition and Suppression of AM Fungi on Plant Community Through Altering Soil Microbes

**Figure 5:**
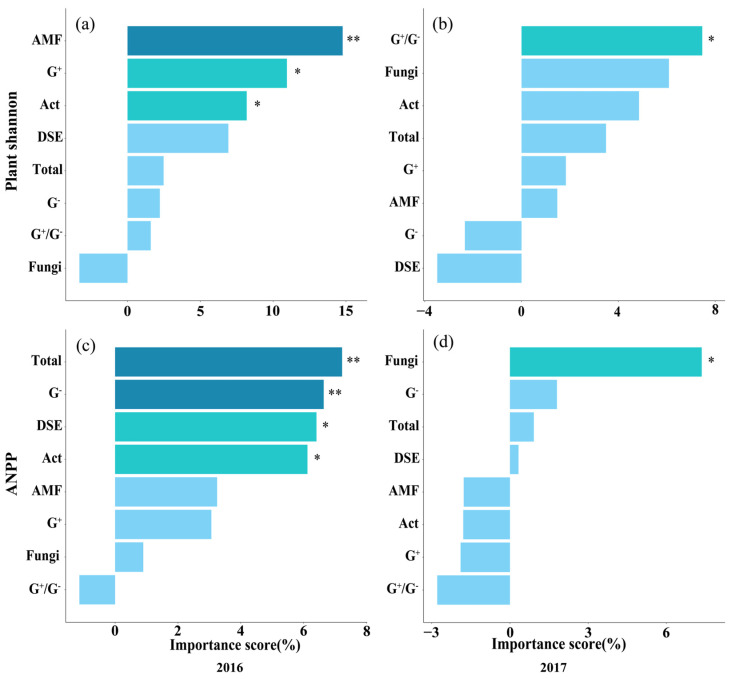
Contribution of soil microbial communities to plant community Shannon diversity and ANPP in 2016 (**a**,**c**) and 2017 (**b**,**d**) based on random forest analysis. Statistical significance was defined as follows: * *p* < 0.05 was considered significant, ** *p* < 0.01 was considered highly significant.

Random forest analysis revealed the relative importance of soil physicochemical properties in driving soil microbial communities (Figure S5), and the relative importance of soil microorganisms in driving plant Shannon diversity and ANPP (Figure 5). The results showed that in the ranking of importance for influencing soil microorganisms, soil NO_3_^−^-N, NH_4_^+^-N, AP, SOC, and pH significantly affected the content of soil microorganisms, including Total, fungi, G^−^, G^+^/G^−^, and DSE (Figure S5). In the ranking of importance for influencing plant Shannon diversity and ANPP, AM fungi, G^+^, and Act significantly affected plant Shannon diversity, while Total microorganisms, G^−^, Act, fungi, and DSE had significant effects on plant ANPP, and the effects were more pronounced in 2016 (Figure 5).

**Figure 6:**
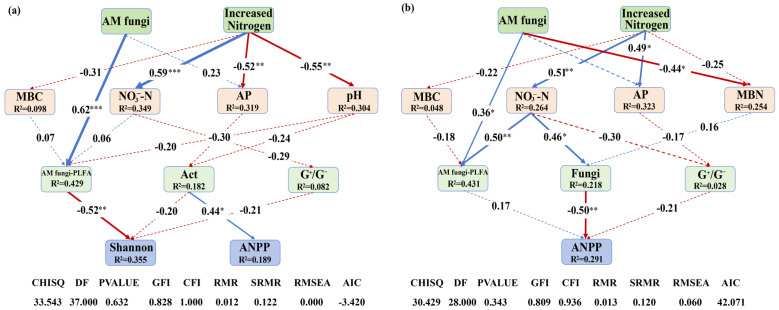
Structural equation modeling (SEM) illustrating causal pathways through which elevated N deposition and AM fungi influence microbial and plant communities. (**a**) SEM results for 2016; (**b**) SEM results for 2017. The blue arrows represent positive effects, the red arrows represent negative effects, and the dashed lines indicate non-significance. Arrow width corresponds to effect strength, with numbers above arrows representing standardized path coefficients. Statistical significance was defined as follows: * *p* < 0.05 was considered significant, ** *p* < 0.01 was considered highly significant, and *** *p* < 0.001 was considered extremely significant. R^2^ values indicate the proportion of variance explained for each dependent variable.

Finally, SEM revealed the impact patterns of increased N deposition and AM fungi on plant Shannon diversity and ANPP by altering soil microorganisms (Figure 6). The SEM results indicated that AM fungi suppression influenced plant Shannon diversity by directly altering the PLFA content of AM fungi in 2016 (Figure 6a). Increased N deposition significantly increased soil NO_3_^−^-N and decreased soil AP and pH, but these properties showed no significant impacts on soil microorganisms in 2016 (Figure 6a). In 2017, increased N deposition significantly increased the NO_3_^−^-N concentrations, which was positively related to soil AM fungi and fungi, and subsequently altered ANPP (Figure 6b). AM fungal suppression influenced ANPP by directly altering the PLFA content of AM fungi (Figure 6b). Overall, these results indicated that increased N deposition and AM fungi altered plant Shannon diversity and ANPP mainly through influencing soil fungi, especially AM fungi.

## 4. Discussion

### 4.1. Potential Influence Pathways of Increased N Deposition and AM Fungi on Soil Microbial Communities

Our findings revealed that increased N deposition posed significant effects on soil microorganisms, promoted the growth of soil G^−^, fungi, and Act, but decreased the G^+^/G^−^ ratio. One previous study demonstrated that increased N deposition promoted total PLFA content of soil microbial community and enhanced the relative abundance of bacteria, fungi, and Act by raising dissolved organic carbon [52]. G^−^ bacteria have been proven to be with higher nutrient use efficiency than G^+^ bacteria [53,54]. Thus, increased N deposition likely promoted the growth of G^−^ bacteria, consequently resulting in the reductions in the G^+^/G^−^ ratio. A study has suggested that increased N deposition could enhance the decomposition of soil organic matter, thereby providing additional carbon substrates for soil microbial growth, which was consistent with our findings [55]. We also found SOC was one of the main influence factors on soil microorganisms, as the increase in SOC enhanced soil fungal growth. In desert ecosystems, soil fungi exhibit a higher tolerance to harsh environment and stronger nutrient responsiveness compared to other soil microorganisms [56]. Thus, the changes in SOC induced by increased N deposition significantly affected soil fungal growth, which was also proved by one previous study in deserts [57]. Furthermore, we found that another factor affecting soil microbial growth was the increase in soil nitrogen content (i.e., NO_3_^−^-N and NH_4_^+^-N) directly resulting from increased N deposition. This phenomenon has also been confirmed in previous studies across different ecosystems. [4]. In desert ecosystems, soil N content is the main limiting resource for soil microbial growth, and the increase in NO_3_^−^-N and NH_4_^+^-N can indeed significantly affect soil microbial communities, particularly fungi [58,59]. Additionally, acidification induced by increased N deposition was the common influence pathway of increased N deposition on soil microorganisms, as we also found based on our results [17]. But it is worth noting that the soils are alkaline and nutrient-poor in desert ecosystems, and soil microorganisms could have been adapted to the harsh environment. Thus, the universal influence mechanisms of increased N deposition on soil microorganisms in desert ecosystems need long-term monitoring in future studies.

Furthermore, AM fungi, as the core soil microbial groups in soil microbial community, play important roles in influencing the growth of soil microorganisms [23]. Our results found that AM fungal suppression significantly increased the growth of soil Act, G^−^, and DSE. This finding corresponds with the research outcomes of one recent study which demonstrated that AM fungal inhibition enhances nutrient accessibility for other microorganisms, consequently elevating PLFA content [60]. The observed discrepancies may stem from the competition between AM fungi and other soil microorganisms for nutrient acquisition. Additionally, our findings revealed that AM fungi significantly reduced the ratios of G^+^/G^−^. Studies have shown that G^−^ generally exhibit greater metabolic flexibility than G^+^, enabling them to utilize nutrient more effectively as an electron acceptor for anaerobic respiration or denitrification [53,54]. Furthermore, our findings indicate that there is a significant interactive effect between AM fungi and increased N deposition on the growth of AM fungi, Act, and fungi. With increasing N deposition, plants not only reduce their reliance on AM fungi for nutrient acquisition but also face constraints in nutrient uptake when these fungi are suppressed [31]. Multiple studies have shown that increased N deposition reduces the abundance of AM fungi, thereby affecting the phospholipid fatty acid content of soil actinomycetes and fungi. [24,30]. Moderate nitrogen addition may promote the growth of Act and increase their PLFA content [55], and increased N deposition reduces the PLFA content of AM fungi. The explanation is that under high N conditions, plants may prefer to directly absorb and utilize soil nitrogen, reducing their reliance on AM fungi, subsequently restricting their growth [61]. Since soil microorganisms depend on root exudates for survival, restricting root activities induced by increased N deposition and AM fungal suppression reduces exudate production, consequently impairing the growth of other soil microorganisms [62].

### 4.2. Increased N Deposition and AM Fungi Altered Plant Community Through Influencing Soil Fungi and AM Fungi

Soil microorganisms play important roles in altering plant community, especially in the face of climate change [9]. Our results indicated that increased N deposition altered the plant community mainly by promoting the growth of soil fungi, especially AM fungi. Increased N deposition could intensify plant–microbial competition for soil nutrient, stimulating plants to enhance the nutrient uptake through microbial mediation and consequently altering the plant community [63]. A two-year nitrogen addition experiment conducted in an arid desert region showed that the soil bacterial alpha diversity initially increased and then decreased with increasing N deposition [15]. Meanwhile, the soil fungal community exhibits higher stability and greater functional resistance to increased N deposition, subsequently leading to changes in the plant community. Furthermore, previous studies demonstrated that G^+^ and G^−^ exhibit distinct ecological functions, demonstrating differential impacts on soil organic matter decomposition, nutrient transformation, and suppression of plant pathogens, and the changes in the G^+^/G^−^ ratio may potentially inhibit plant growth [64], while our study indicated that soil fungi, particularly AM fungi, play key roles in altering the plant community under increased N deposition in desert ecosystems. Two factors might explain this result. First, soil fungi have been proven to be more tolerant to environmental perturbations than other soil microorganisms, especially in barren environments [12]. Second, AM fungi can form symbionts with more than 80% terrestrial plants, and more than 90% desert plants can form symbionts with AM fungi [29]. This indicated that AM fungi play important roles in maintaining the plant community diversity, biomass, nutrient cycling and other ecological functions in deserts, which have been widely demonstrated in previous studies [6,9].

AM fungi directly alter the plant community due to the close symbiotic relationships with plants [29]. AM fungi can form a symbiotic relationship with plant roots, enhancing the plant’s absorption of water and nutrients, and this effect is particularly significant in impoverished desert soils. [65]. Research has demonstrated positive correlations between soil fungal diversity and above-ground plant diversity in grassland ecosystems [66]. The reduction in AM fungal abundance impaired plant root growth, consequently leading to declines in both the plant community diversity and evenness [67]. Our results confirmed this finding and further indicated that AM fungi would play more important roles in drought conditions. Our analysis suggested that plant species exhibit differential drought adaptability, as plants which rely on AM fungi for nutrient acquisition and drought tolerance may increase their growth under environmental perturbations, such as drought, and ultimately alter plant community structure. Furthermore, our results found that increased N deposition in conjunction with AM fungi elevated ANPP by enhancing the activity of both soil fungi and AM fungi. A possible explanation is that AM fungi play a crucial role in plant communities, and their suppression altered plant strategies for nutrient and water acquisition, thereby affecting plant community structure [68]. Increased N deposition may alter soil nutrient availability, affect plant growth and competitive interactions [69]. Following AM fungal suppression, although increased N deposition could enrich soil nutrients, the restricted nutrient uptake capacity due to impaired AM fungi ultimately caused significant changes in the plant community [70]. It is worth to note that soil fungi and other microorganisms could have been adapted to the barren environments with poor nutrients. The long-term increased N deposition could lead to a simplification of soil microbial communities, consequently diminish their selectivity towards different plant species and ultimately resulting in the declines in their ecological functions [71]. Thus, future studies considering the roles of soil microorganisms in altering the plant community should be taken into account to compare the N deposition effects on the plant community across diverse ecosystems.

## 5. Conclusions

In summary, this paper reveals the impact patterns of AM fungi on soil microorganisms under the context of N deposition and estimates the effects of soil microorganisms on plant communities in desert ecosystems. We found that increased N deposition clearly promoted soil AM fungi, fungi, Act, G^+^ and G^−^. AM fungal suppression significantly increased the content of soil Act and G^+^. And, there were clearly and significantly interactive effects of increased N deposition and AM fungi on soil microbial contents. Increased N deposition significantly increased the growth of soil fungi and Act when AM fungi were suppressed, while it posed limited effects when AM fungi were present. Increased N deposition significantly increased the growth of G^+^ and G^−^ when AM fungi were present, while no significant effect was observed when AM fungi were suppressed. Both increased N deposition and AM fungi caused significant changes in soil microbial community structure. Random forest analysis indicated that increased N deposition affects soil microorganisms by elevating soil NH_4_^+^-N and NO_3_^−^-N content. SEM analysis revealed that increased N deposition elevates the abundance of soil fungi and AM fungi, by increasing NO_3_^−^-N levels. We found that increased N deposition reduces plant Shannon diversity, and the presence of AM fungi can mitigate this negative effect. Finally, SEM results indicated that soil fungi, especially AM fungi, were the main soil microorganisms altering the plant community diversity and ANPP under increased N deposition. In summary, increased N deposition affects soil microorganisms by elevating soil NO_3_^−^-N levels and reducing pH, and these effects manifest differently depending on the presence or suppression of AM fungi. Follow-up studies could incorporate metagenomics to elucidate the underlying mechanisms and other potential impacts.

## Figures and Tables

**Table 1:** The effects of increased nitrogen deposition and AM fungal suppression on soil physicochemical properties (2016 and 2017). All data are presented as “mean ± standard error”. Results of the ANOVA are detailed in Supplementary Table S1.

Year	Treatment	pH	AP	SOC	NO_3_^−^-N	NH_4_^+^-N	MBC	MBN
			(mg/kg)	(g/kg)	(mg/kg)	(mg/kg)	(mg/kg)	(mg/kg)
2016	W	8.60 ± 0.14 a	6.65 ± 0.49 a	2.11 ± 0.02 b	6.29 ± 0.17 b	1.6 ± 0.08 c	165.80 ± 5.53 a	65.48 ± 0.70 a
	BW	8.59 ± 0.15 a	6.37 ± 0.46 a	2.17 ± 0.08 b	6.48 ± 0.18 ab	2.12 ± 0.14 b	168.86 ± 4.93 a	65.76 ± 1.10 a
	NW	8.13 ± 0.09 b	5.74 ± 0.20 ab	2.45 ± 0.11 a	6.94 ± 0.19 a	2.74 ± 0.18 a	156.29 ± 4.5a	62.81 ± 0.43 b
	BNW	8.32 ± 0.14 ab	5.04 ± 0.51 b	2.25 ± 0.13 ab	6.89 ± 0.16 a	2.61 ± 0.19 a	166.79 ± 2.11 a	65.37 ± 0.25 a
2017	W	8.54 ± 0.08 a	4.38 ± 0.45 ab	2.13 ± 0.02 b	6.44 ± 0.16 b	1.167 ± 0.04 c	168.02 ± 5.42 a	64.91 ± 1.03 ab
	BW	8.39 ± 0.16 a	3.77 ± 0.38 b	2.21 ± 0.09 ab	6.50 ± 0.18 ab	1.52 ± 0.11 b	173.10 ± 5.20 a	65.89 ± 1.04 a
	NW	8.04 ± 0.02 b	5.42 ± 0.36 b	2.33 ± 0.06 a	6.90 ± 0.16 a	2.82 ± 0.14 a	169.82 ± 5.59 a	62.58 ± 0.83 b
	BNW	8.31 ± 0.16 ab	4.82 ± 0.51 ab	2.17 ± 0.08 ab	6.90 ± 0.2 a	2.07 ± 0.04 a	161.48 ± 3.95 a	65.84 ± 1.01 a

Note: Different lowercase letters (a, b, c) indicate significant differences among treatments (*p* < 0.05). W, water treatment; BW, benomyl + water treatment; NW, N + water treatment; BNW, benomyl + N + water treatment; AP, Available phosphorus; SOC, Soil organic carbon; NO_3_^−^-N, Nitrate nitrogen; NH_4_^+^-N, Ammonium nitrogen; MBC, Microbial biomass carbon; MBN, Microbial biomass nitrogen.

## Data Availability

The original contributions presented in this study are included in the article/Supplementary Materials. Further inquiries can be directed to the corresponding author.

## Supplementary Materials

The following supporting information can be downloaded at https://www.mdpi.com/article/10.3390/microorganisms13122660/s1, Table S1: Results of two-way factorial analysis of variance (ANOVA) on the effects of increased nitrogen deposition (N) and suppression of Arbuscular mycorrhiza (AM) fungi (B) on soil physicochemical properties in 2016 and 2017; Table S2: Results of two-factor ANOVA on plant phospholipid fatty acid (PLFA) content under increased N deposition and suppression of AM fungi in 2016 and 2017; Table S3: Results of two-factor ANOVA on soil microbial PLFA content under increased N deposition and suppression of AM fungi in 2016 and 2017; Table S4: Results of two-factor ANOVA on soil microbial diversity under increased N deposition and suppression of AM fungi in 2016 and 2017; Figure S1: Effects of increased nitrogen deposition (N) and suppression of Arbuscular mycorrhiza (AM) fungi on spore density and hyphal density content under different treatments; Figure S2: Effects of increased nitrogen deposition and AM fungi on the relative abundance of various soil microbial groups were observed in 2016 and 2017, respectively; Figure S3: Effects of increased N deposition and AM fungi on soil microbial communities in 2016 and 2017 based on non-metric multidimensional scaling (NMDS) analysis; Figure S4: Redundancy Analysis (RDA) of Physicochemical Properties, Plants, and Microbes; Figure S5: Contribution of soil physicochemical properties to soil microorganisms in 2016 and 2017 based on random forest analysis.

## References

[1] La Sorte F.A., Johnston A., Ault T.R. (2021). Global Trends in the Frequency and Duration of Temperature Extremes. Clim. Change.

[2] LeBauer D.S., Treseder K.K. (2008). Nitrogen Limitation of Net Primary Productivity in Terrestrial Ecosystems Is Globally Distributed. Ecology.

[3] Tian D., Niu S. (2015). A Global Analysis of Soil Acidification Caused by Nitrogen Addition. Environ. Res. Lett..

[4] Lu X., Mao Q., Gilliam F.S., Luo Y., Mo J. (2014). Nitrogen Deposition Contributes to Soil Acidification in Tropical Ecosystems. Glob. Change Biol..

[5] Zhou C., Zuo S., Wang X., Ji Y., Lamao Q., Liu L., Huang D. (2022). Effects of Grazing Sheep and Mowing on Grassland Vegetation Community and Soil Microbial Activity under Different Levels of Nitrogen Deposition. Agriculture.

[6] Iqbal S., Begum F., Nguchu B.A., Claver U.P., Shaw P. (2025). The invisible Architects: Microbial Communities and Their Transformative Role in Soil Health and Global Climate Changes. Environ. Microbiome.

[7] Li W., Xie L., Zhao C., Hu X., Yin C. (2023). Nitrogen Fertilization Increases Soil Microbial Biomass and Alters Microbial Composition Especially Under Low Soil Water Availability. Microb. Ecol..

[8] Gallardo A., Schlesinger W.H. (1992). Carbon and Nitrogen Limitations of Soil Microbial Biomass in Desert Ecosystems. Biogeochemistry.

[9] Kowalchuk G.A., Buma D.S., de Boer W., Klinkhamer P.G., van Veen J.A. (2002). Effects of above-Ground Plant Species Composition and Diversity on the Diversity of Soil-Borne Microorganisms. Antonie Van Leeuwenhoek.

[10] Roth T., Kohli L., Rihm B., Achermann B. (2013). Nitrogen Deposition Is Negatively Related to Species Richness and Species Composition of Vascular Plants and Bryophytes in Swiss Mountain Grassland. Agric. Ecosyst. Environ..

[11] Shen H., Dong S., DiTommaso A., Xiao J., Lu W., Zhi Y. (2022). Nitrogen Deposition Shifts Grassland Communities Through Directly Increasing Dominance of Graminoids: A 3-Year Case Study from the Qinghai-Tibetan Plateau. Front. Plant Sci..

[12] She W., Bai Y., Zhang Y., Qin S., Feng W., Sun Y., Zheng J., Wu B. (2018). Resource Availability Drives Responses of Soil Microbial Communities to Short-term Precipitation and Nitrogen Addition in a Desert Shrubland. Front. Microbiol..

[13] Xi D., Jin S., Wu J. (2022). Soil Bacterial Community Is More Sensitive Than Fungal Community to Canopy Nitrogen Deposition and Understory Removal in a Chinese Fir Plantation. Front. Microbiol..

[14] Zhang X., Song X., Wang T., Huang L., Ma H., Wang M., Tan D. (2022). The Responses to Long-Term Nitrogen Addition of Soil Bacterial, Fungal, and Archaeal Communities in a Desert Ecosystem. Front. Microbiol..

[15] Zhang Z., Tang G., Chai X., Liu B., Gao X., Zeng F., Wang Y., Zhang B. (2023). Different Responses of Soil Bacterial and Fungal Communities in Three Typical Vegetations following Nitrogen Deposition in an Arid Desert. Microorganisms.

[16] Jörgensen K., Clemmensen K.E., Wallander H., Lindahl B.D. (2024). Ectomycorrhizal Fungi Are More Sensitive to High Soil Nitrogen Levels in Forests Exposed to Nitrogen Deposition. New Phytol..

[17] Liu J., Zhu M., Shi X., Hui C., Sun Y., Zhang R., Jin D., Li Z., Chen H., Zhao Z. (2024). Cascading Impacts of Nitrogen Deposition on Soil Microbiome and Herbivore Communities in Desert Steppes. Sci. Total Environ..

[18] Song B., Li Y., Yang L., Shi H., Li L., Bai W., Zhao Y. (2023). Soil Acidification Under Long-Term N Addition Decreases the Diversity of Soil Bacteria and Fungi and Changes Their Community Composition in a Semiarid Grassland. Microb. Ecol..

[19] Jiang N., Zhang H., Zhang S., Qin J., Wang H., Zhang Y., Yang D., Wang L., Yang Q., Ye H. (2025). Different Responses of Soil Bacterial Necromass Carbon and Fungal Necromass Carbon to Nitrogen Deposition in Meadow Steppe. Appl. Soil Ecol..

[20] Zhao Y., Chen H., Sun H., Yang F. (2024). In the Qaidam Basin, Soil Nutrients Directly or Indirectly Affect Desert Ecosystem Stability under Drought Stress through Plant Nutrients. Plants.

[21] Lu G., Xie B., Cagle G.A., Wang X., Han G., Wang X., Hou A., Guan B. (2021). Effects of Simulated Nitrogen Deposition on Soil Microbial Community Diversity in Coastal Wetland of the Yellow River Delta. Sci. Total Environ..

[22] Belov A.A., Cheptsov V.S., Vorobyova E.A., Manucharova N.A., Ezhelev Z.S. (2019). Stress-Tolerance and Taxonomy of Culturable Bacterial Communities Isolated from a Central Mojave Desert Soil Sample. Geosciences.

[23] Horn S., Hempel S., Verbruggen E., Rillig M.C., Caruso T. (2017). Linking the Community Structure of Arbuscular Mycorrhizal Fungi and Plants: A Story of Interdependence?. ISME J..

[24] Verbruggen E., Van Der Heijden M.G., Weedon J.T., Kowalchuk G.A., Röling W.F. (2012). Community Assembly, Species Richness and Nestedness of Arbuscular Mycorrhizal Fungi in Agricultural Soils. Mol. Ecol..

[25] Fan L., Zhang P., Cao F., Liu X., Ji M., Xie M. (2024). Effects of AMF on Maize Yield and Soil Microbial Community in Sandy and Saline Soils. Plants.

[26] Gou X., Kong W., Sadowsky M.J., Chang X., Qiu L., Liu W., Shao M., Wei X. (2024). Global Responses of Soil Bacteria and Fungi to Inoculation With Arbuscular Mycorrhizal Fungi. Catena.

[27] Yang N., Zhang J., Hua J., Song B., Wang T., Xing W., Wang G., Mao L., Ruan H. (2024). Differentiation of Fungal Trophic Guilds to Long-Term Nitrogen Addition in a Poplar Plantation. For. Ecol. Manag..

[28] Pozo M.J., Azcón-Aguilar C. (2007). Unraveling Mycorrhiza-Induced Resistance. Curr. Opin. Plant Biol..

[29] Duan S., Feng G., Limpens E., Bonfante P., Xie X., Zhang L. (2024). Cross-Kingdom Nutrient Exchange in the Plant-Arbuscular Mycorrhizal Fungus-Bacterium Continuum. Nat. Rev. Microbiol..

[30] Han Y., Feng J., Han M., Zhu B. (2020). Responses of Arbuscular Mycorrhizal Fungi to Nitrogen Addition: A Meta-Analysis. Glob. Change Biol..

[31] Zhai C., Yang Y., Kong L., Wang X., Hou J., Zeng Q., Ge A., Yao B., Zhou Z., Feng J. (2025). Nitrogen Deposition Decouples Grassland Plant Community From Soil Bacterial and Fungal Communities along a Precipitation Gradient. J. Ecol..

[32] Ezcurra E., Mellink E. (2013). Desert Ecosystems. Encyclopedia of Biodiversity.

[33] Han J., Wang J., Zhao C., Yue C., Liu Z. (2025). Desertification Dynamics and Future Projections in Qaidam Basin, China. Environ. Monit. Assess..

[34] Yu X., Lei J., Gao X. (2022). An Over Review of Desertification in Xinjiang, Northwest China. J. Arid Land.

[35] Bai Y., Wu J., Xing Q., Pan Q., Huang J., Yang D., Han X. (2008). Primary Production and Rain Use Efficiency across a Precipitation Gradient on the Mongolia Plateau. Ecology.

[36] Liu Y., Li X., Zhang Q., Guo Y., Gao G., Wang J. (2010). Simulation of regional Temperature and Precipitation in the Past 50 Years and the Next 30 Years over China. Quat. Int..

[37] Zhou X., Zhang Y., Niklas K.J. (2014). Sensitivity of Growth and Biomass Allocation Patterns to Increasing Nitrogen: A Comparison Between Ephemerals and Annuals in the Gurbantunggut Desert, North-Western China. Ann. Bot..

[38] Cui X., Yue P., Gong Y., Li K., Tan D., Goulding K., Liu X. (2017). Impacts of Water and Nitrogen Addition on Nitrogen Recovery in Haloxylon Ammodendron Dominated Desert Ecosystems. Sci. Total Environ..

[39] Zhao R., Hui R., Liu L., Xie M., An L. (2018). Effects of Snowfall Depth on Soil Physical–Chemical Properties and Soil Microbial Biomass in Moss–Dominated Crusts in the Gurbantunggut Desert, Northern China. Catena.

[40] Wang X., Jiang J., Wang Y., Luo W., Song C., Chen J. (2006). Responses of Ephemeral Plant Germination and Growth to Water and Heat Conditions in the Southern Part of Gurbantunggut Desert. Chin. Sci. Bull..

[41] Meng H., Zhang Y., Zhou X., Yin B., Zhou D., Tao Y. (2022). Biomass Allocation Patterns of Herbaceous Plants in the Gurbantunggut Desert. J. Desert Res..

[42] Zhao W.Q., Lv X.H., Li Y.G., Wang Z.K., Zhang W., Zhuang L. (2019). Future N Deposition and Precipitation Changes Will Be Beneficial for the Growth of Haloxylon Ammodendron In Gurbantunggut Desert, northwest China. Sci. Rep..

[43] Hartnett D.C., Wilson G.W.T. (1999). Mycorrhizae Influence Plant Community Structure and Diversity in Tallgrass Prairie. Ecology.

[44] O’Connor P.J., Smith S.E., Smith F.A. (2002). Arbuscular Mycorrhizas Influence Plant Diversity and Community Structure in a Semiarid Herbland. New Phytol..

[45] Yang G., Liu N., Lu W., Wang S., Kan H., Zhang Y., Xu L., Chen Y. (2014). The Interaction Between Arbuscular Mycorrhizal Fungi and Soil Phosphorus Availability Influences Plant Community Productivity and Ecosystem Stability. J. Ecol..

[46] Galloway J.N. (2005). The Global Nitrogen Cycle: Past, Present and Future. Sci. China Life Sci..

[47] Zelles L. (1997). Phospholipid Fatty Acid Profiles in Selected Members of Soil Microbial Communities. Chemosphere.

[48] Bossio D.A., Scow K.M. (1998). Impacts of Carbon and Flooding on Soil Microbial Communities: Phospholipid Fatty Acid Profiles and Substrate Utilization Patterns. Microb. Ecol..

[49] White D.C., Flemming C.A., Leung K.T., Macnaughton S.J. (1998). In Situ Microbial Ecology for Quantitative Appraisal, Monitoring, and Risk Assessment of Pollution Remediation in Soils, the Subsurface, the Rhizosphere and in Biofilms. J. Microbiol. Methods.

[50] Bardgett R.D. (1991). The Use of the Membrane Filter Technique for Comparative Measurements of Hyphal Lengths in Different Grassland Sites. Agric. Ecosyst. Environ..

[51] Tamir H., Gilvarg C. (1966). Density Gradient Centrifugation for the Separation of Sporulating Forms of Bacteria. J. Biol. Chem..

[52] Lv F., Xue S., Wang G., Zhang C. (2017). Nitrogen Addition Shifts the Microbial Community in the Rhizosphere of Pinus Tabuliformis in Northwestern China. PLoS ONE.

[53] Amon J., Titgemeyer F., Burkovski A. (2010). Common Patterns—Unique features: Nitrogen Metabolism and Regulation in Gram-Positive Bacteria. FEMS Microbiol. Rev..

[54] Liu X., Wang Q., Li L., Sun X., Lv A., Chen C. (2020). Characterization of Aerobic Denitrification Genome Sequencing of Vibrio Parahaemolyticus Strain Ha2 from Recirculating Mariculture System in China. Aquaculture.

[55] Li S., Lyu M., Deng C., Deng W., Wang X., Cao A., Jiang Y., Liu J., Lu Y., Xie J. (2024). Input of High-Quality Litter Reduces Soil Carbon Losses Due to Priming in a Subtropical Pine Forest. Soil Biol. Biochem..

[56] Hamm P.S., Mueller R.C., Kuske C.R., Porras-Alfaro A. (2020). Keratinophilic Fungi: Specialized Fungal Communities in a Desert Ecosystem Identified Using Cultured-Based and Illumina Sequencing Approaches. Microbiol. Res..

[57] Zuo Y., He C., Zhang D., Zhao L., He X., Sun X. (2023). Soil Variables Driven by Host Plant and Growth Season Affect Soil Microbial Composition and Metabolism in Extremely Arid Desert Ecosystems. Microbiol. Res..

[58] Jianbo W., Na L., Xiaodan W. (2020). Nitrogen Deposition Strengthens the Relationship Between Plants and the Soil Fungal Community In Alpine Steppe, North Tibet. Appl. Soil Ecol..

[59] Xing A., Xu L., Zhao M., Shen H., Ma S., Fang J. (2022). Shifts in Understory Plant Composition Induced by Nitrogen Addition Predict Soil Fungal Beta Diversity in a Boreal Forest. Biol. Fertil. Soils.

[60] Canarini A., Schmidt H., Fuchslueger L., Martin V., Herbold C.W., Zezula D., Gündler P., Hasibeder R., Jecmenica M., Bahn M. (2021). Ecological Memory of Recurrent Drought Modifies Soil Processes Via Changes In Soil Microbial Community. Nat. Commun..

[61] Bahadur A., Jin Z., Long X., Jiang S., Zhang Q., Pan J., Liu Y., Feng H. (2019). Arbuscular Mycorrhizal Fungi Alter Plant Interspecific Interaction under Nitrogen Fertilization. Eur. J. Soil Biol..

[62] Bogati K., Walczak M. (2022). The Impact of Drought Stress on Soil Microbial Community, Enzyme Activities and Plants. Agronomy.

[63] Bi J., Zhang N.L., Liang Y., Yang H.J., Ma K.P. (2012). Interactive Effects of Water and Nitrogen Addition on Soil Microbial Communities in a Semiarid Steppe. J. Plant Ecol..

[64] Thapa S.P., Davis E.W., Lyu Q., Weisberg A.J., Stevens D.M., Clarke C.R., Coaker G., Chang J.H. (2019). The Evolution, Ecology, and Mechanisms of Infection by Gram-Positive, Plant-Associated Bacteria. Annu. Rev. Phytopathol..

[65] Kumar U., Raj S., Sreenikethanam A., Maddheshiya R., Kumari S., Han S., Kapoor K.K., Bhaskar R., Bajhaiya A.K., Gahlot D.K. (2023). Multi-Omics Approaches in Plant–Microbe Interactions Hold Enormous Promise for Sustainable Agriculture. Agronomy.

[66] Wang C., Ma L., Zuo X., Ye X., Wang R., Huang Z., Liu G., Cornelissen J.H.C. (2022). Plant Diversity Has Stronger Linkage with Soil Fungal Diversity than with Bacterial Diversity across Grasslands of Northern China. Glob. Ecol. Biogeogr..

[67] Ma X., Geng Q., Zhang H., Bian C., Chen H.Y.H., Jiang D., Xu X. (2021). Global Negative Effects of Nutrient Enrichment on Arbuscular Mycorrhizal Fungi, Plant Diversity and Ecosystem Multifunctionality. New Phytol..

[68] Ji Z., Dong Q., Yang R., Qin W., Peng Y., Jia Y. (2025). From Ordinary to Extraordinary: The Crucial Role of Common Species in Desert Plant Community Stability with Arbuscular Mycorrhizal (AM) Fungi Under Increased Precipitation. Plants.

[69] Ma Q., Liu X., Li Y., Li L., Yu H., Qi M., Zhou G., Xu Z. (2020). Nitrogen Deposition Magnifies the Sensitivity of Desert Steppe Plant Communities to Large Changes in Precipitation. J. Ecol..

[70] Stanescu S., Maherali H. (2017). Arbuscular Mycorrhizal Fungi Alter the Competitive Hierarchy among Old-Field Plant Species. Oecologia.

[71] Guzman A., Montes M., Hutchins L., DeLaCerda G., Yang P., Kakouridis A., Dahlquist-Willard R.M., Firestone M.K., Bowles T., Kremen C. (2021). Crop Diversity Enriches Arbuscular Mycorrhizal Fungal Communities in an Intensive Agricultural Landscape. New Phytol..

