# Supplementary material for: Influence Pattern and Mechanism of Increased Nitrogen Deposition and AM Fungi on Soil Microbial Community in Desert Ecosystems
Source: Microorganisms. 2025 Nov 22;13(12):2660. doi: 10.3390/microorganisms13122660 (PMC12734505; doi:10.3390/microorganisms13122660)
Supplement: Supplementary file 1 [file microorganisms-13-02660-s001.zip › microorganisms-3901629-supplementary.pdf]

Table S1. Results of two-way factorial analysis of variance (ANOVA) on the effects of increased nitrogen deposition (N) and suppression of Arbuscular mycorrhiza (AM) fungi (B) on soil physicochemical properties in 2016 and 2017. p values in bold indicate significant differences (p < 0.05).

Year	Treatment	pH	AP	SOC	NO_3^--N	NH_4^+-N	MBC	MBN
2016	B	0.842	0.208	0.316	0.198	**p < 0.001**	0.949	0.777
	N	**p < 0.05**	0.159	**p < 0.05**	**p < 0.05**	**p < 0.001**	0.409	**p < 0.05**
	B*N	0.102	0.346	**p < 0.05**	0.060	**p < 0.001**	0.498	**p < 0.05**
2017	B	0.178	0.224	0.520	0.487	0.810	0.915	0.426
	N	**p < 0.05**	0.053	**p < 0.05**	0.063	**p < 0.001**	0.799	0.081
	B*N	**p < 0.05**	0.514	0.057	0.224	**p < 0.001**	0.634	0.111

Note: AP, Available phosphorus; SOC, Soil organic carbon; NO_3^--N, Nitrate nitrogen; NH_4^+-N, Ammonium nitrogen; MBC, Microbial biomass carbon; MBN, Microbial biomass nitrogen.

Table S2. Results of two-factor ANOVA on plant phospholipid fatty acid (PLFA) content under increased N deposition and suppression of AM fungi in 2016 and 2017.

Year	Treatment	Shannon	Evenness	Richness	ANPP	coverage (%/m^2)	density (NO./m^2)
2016	B	0.155	0.247	0.534	**$p < 0.05$**	0.962	0.409
	N	0.942	0.952	0.678	0.112	**$p < 0.001$**	**$p < 0.001$**
	B*N	0.382	0.461	0.678	**$p < 0.05$**	0.478	0.385
2017	B	0.279	**$p < 0.001$**	0.848	0.334	0.097	**$p < 0.05$**
	N	0.754	**$p < 0.001$**	0.136	0.469	**$p < 0.001$**	**$p < 0.05$**
	B*N	0.591	**$p < 0.05$**	0.343	0.060	0.494	0.053

Table S3. Results of two-factor ANOVA on soil microbial PLFA content under increased N deposition and suppression of AM fungi in 2016 and 2017.

Year	Treatment	Total (nmol/g)	AMF (nmol/g)	Fungi (nmol/g)	Act (nmol/g)	G^+ (nmol/g)	G^- (nmol/g)	G^+/G^-	DSE (nmol/g)
2016	B	0.259	**$p < 0.001$**	0.814	0.354	0.224	0.156	0.894	0.499
	N	0.117	0.123	**$p < 0.05$**	**$p < 0.001$**	0.344	0.056	**$p < 0.05$**	0.072
	B*N	0.052	0.633	**$p < 0.05$**	**$p < 0.001$**	**$p < 0.05$**	0.793	**$p < 0.05$**	**$p < 0.05$**
2017	B	**$p < 0.05$**	0.062	0.174	**$p < 0.001$**	**$p < 0.001$**	0.137	**$p < 0.05$**	0.754
	N	**$p < 0.001$**	**$p < 0.05$**	**$p < 0.05$**	**$p < 0.001$**	**$p < 0.05$**	**$p < 0.05$**	0.428	**$p < 0.001$**
	B*N	**$p < 0.05$**	0.211	**$p < 0.05$**	**$p < 0.05$**	**$p < 0.05$**	**$p < 0.05$**	0.141	0.337

Note: Total, Total soil microbial biomass; AM Fungi, Arbuscular mycorrhizal fungi; Act, Actinomycetes; G^+, Gram-positive bacteria; G^-, Gram-negative bacteria; G^+/G^-, Ratio of Gram-positive to Gram-negative bacteria; DSE, Dark septate endophytes.

Table S4. Results of two-factor ANOVA on soil microbial diversity under increased N deposition and suppression of AM fungi in 2016 and 2017.

Year	Treatment	Micro-Shannon	Micro-simspon	Micro-pielou
2016	B	**$p < 0.05$**	0.053	**$p < 0.05$**
	N	0.752	0.568	0.752
	B*N	0.053	0.052	0.053
2017	B	**$p < 0.05$**	**$p < 0.001$**	**$p < 0.05$**
	N	0.168	**$p < 0.001$**	0.168
	B*N	**$p < 0.05$**	0.998	**$p < 0.05$**

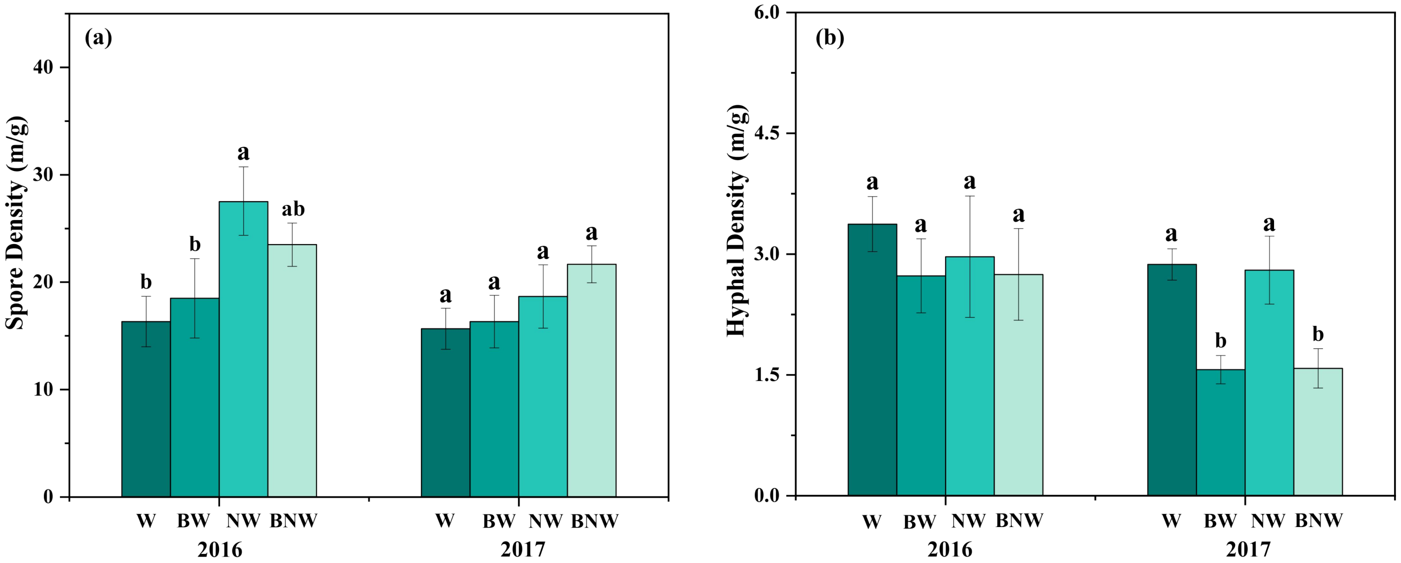

Figure S1. Effects of increased nitrogen deposition (N) and suppression of Arbuscular mycorrhiza (AM) fungi on spore density and hyphal density content under different treatments.

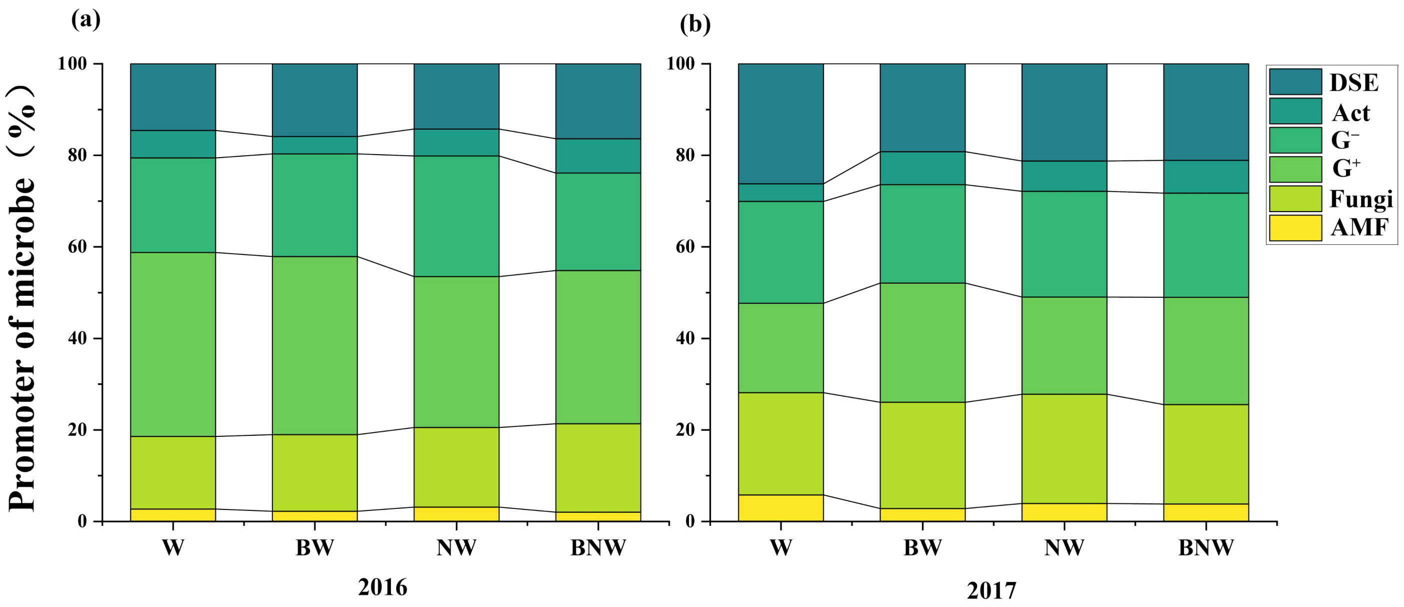

Figure S2. Effects of increased nitrogen deposition and AM fungi on the relative abundance of various soil microbial groups were observed in 2016 and 2017, respectively. Note: W, water treatment; BW, benomyl + water treatment; NW, N + water treatment; BNW, benomyl + N + water treatment； Total, Total soil microbial biomass; AM Fungi, Arbuscular mycorrhizal fungi; Act, Actinomycetes; G^+, Gram-positive bacteria; G^-, Gram-negative bacteria; G^+/G^-, Ratio of Gram-positive to Gram-negative bacteria; DSE, Dark septate endophytes.

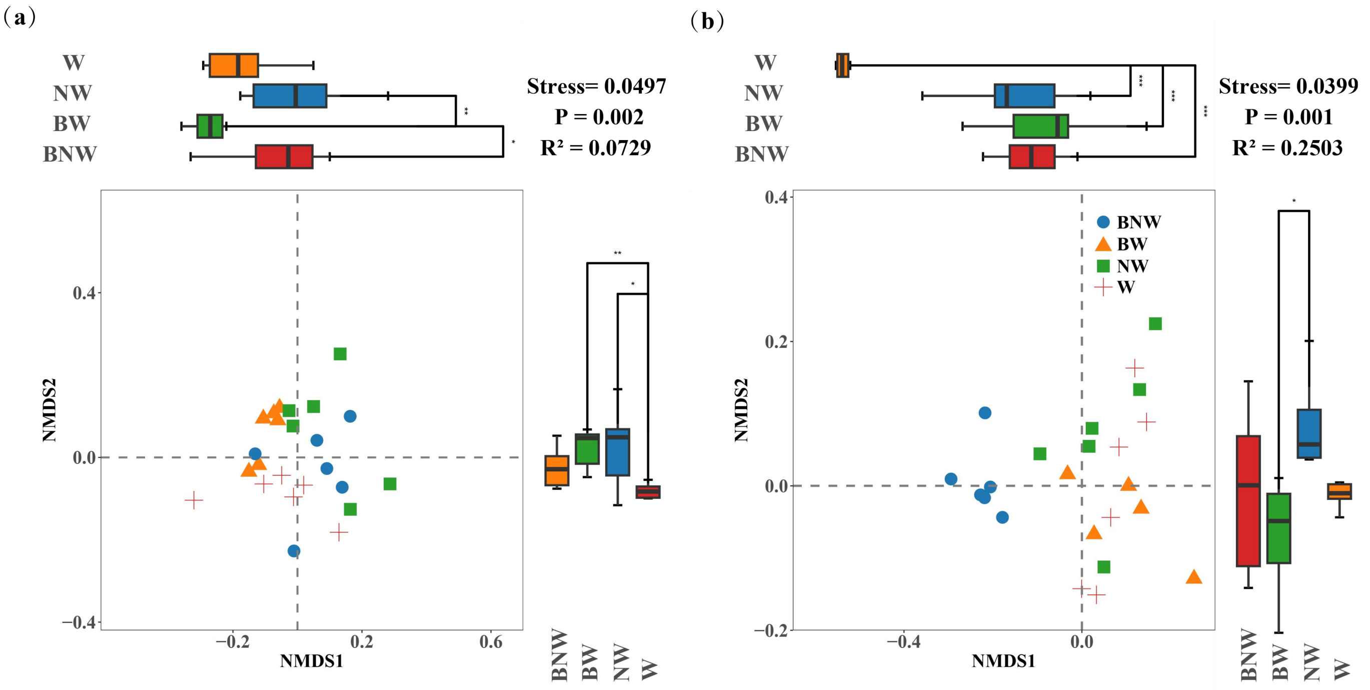

Figure S3. Effects of increased N deposition and AM fungi on soil microbial communities in 2016 and 2017 based on non-metric multidimensional scaling (NMDS) analysis.

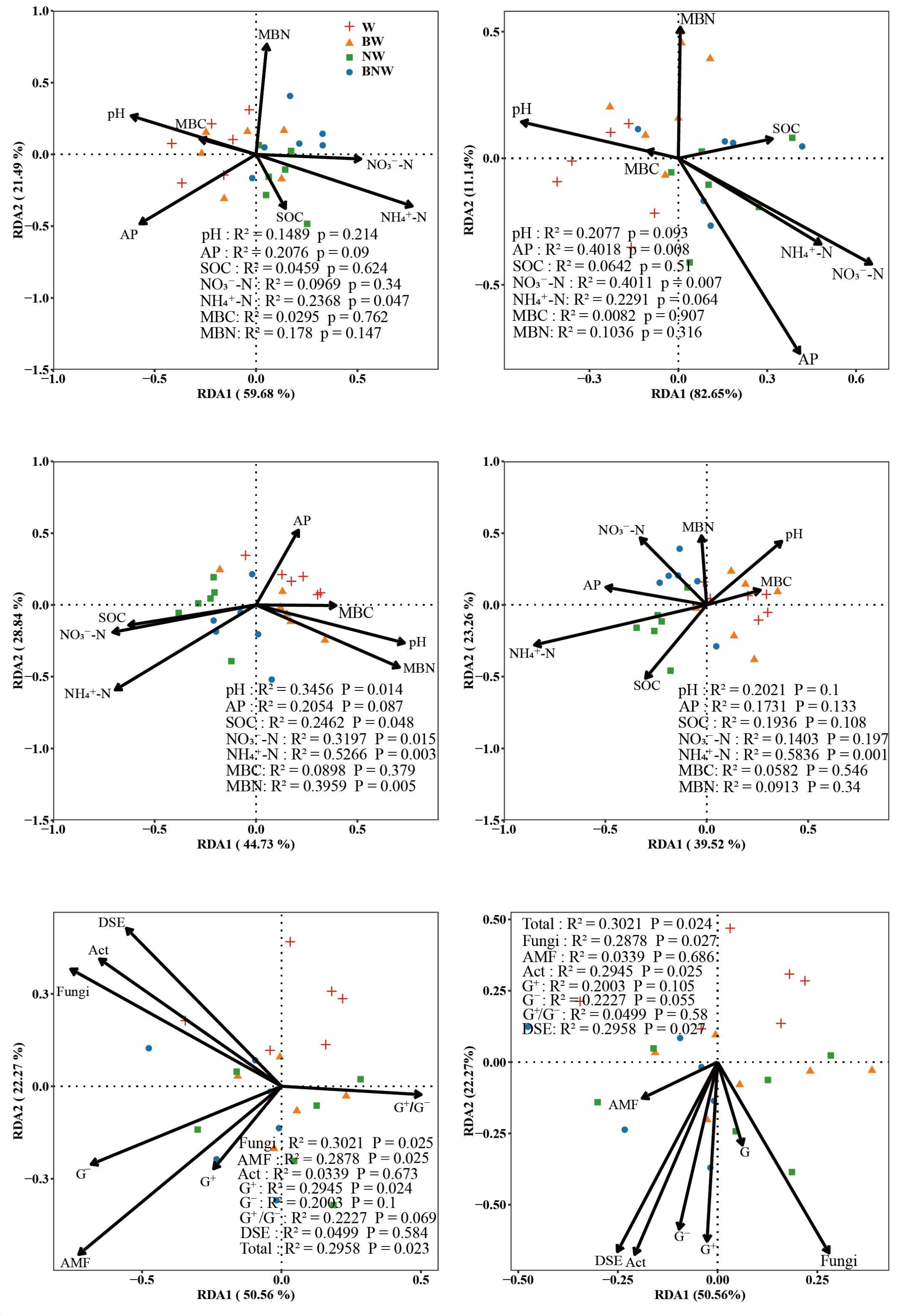

Figure S4. Redundancy Analysis（RDA） of Physicochemical Properties, Plants, and Microbes. (a) RDA of Physicochemical Factors and Microbes in 2016; (b) RDA of Physicochemical Factors and Microbes in 2017; (c) RDA of Physicochemical Factors and Plants in 2016; (d) RDA of Physicochemical Factors and Plants in 2017; (e) RDA of Microbes and Plants in 2016; (f) RDA of Microbes and Plants in 2017.

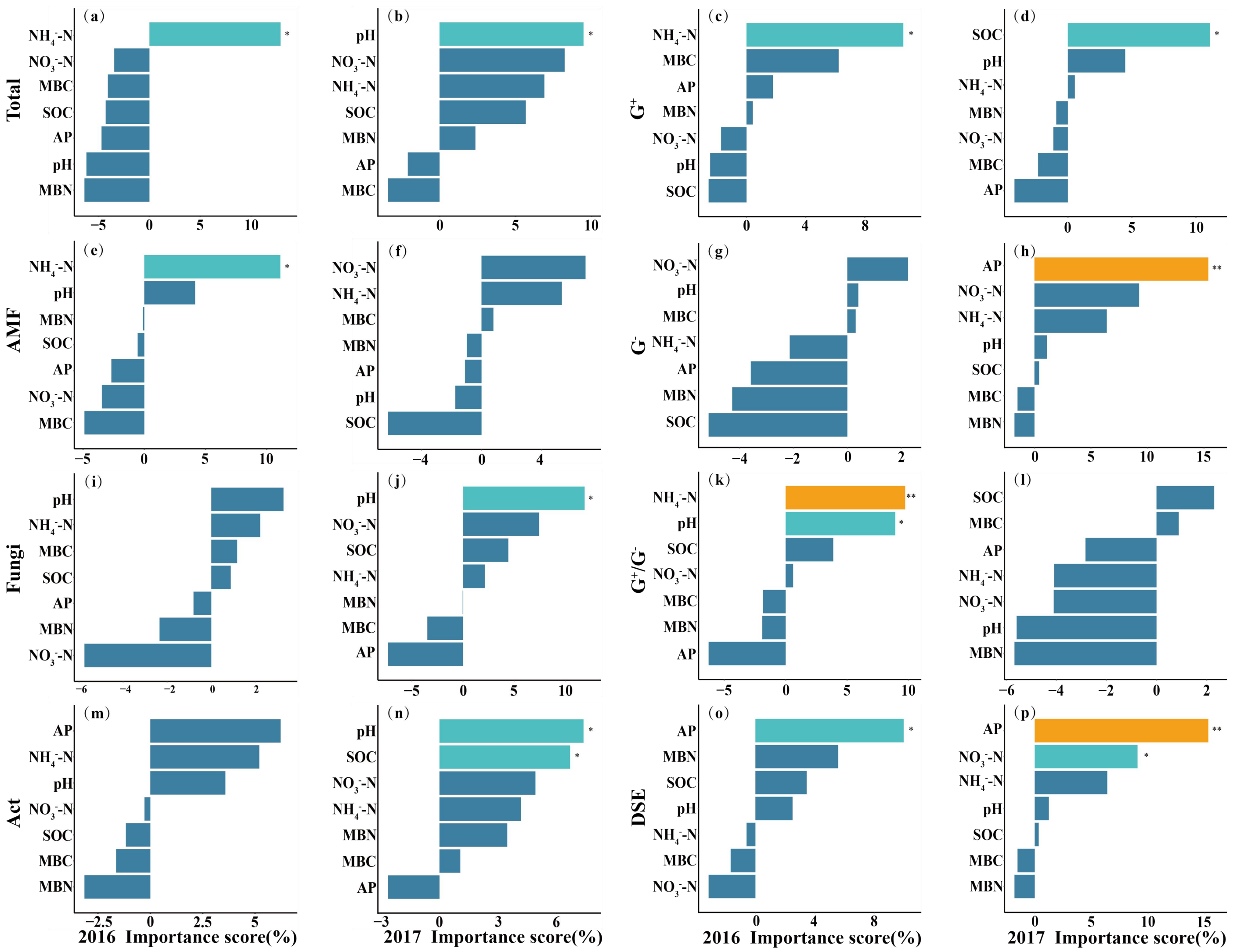

Figure S5. Contribution of soil physicochemical properties to soil microorganisms in 2016 and 2017 based on random forest analysis. *, $p < 0.05$; **, $p < 0.01$.
